# Efficacy and Safety of Ashwagandha (Withania somnifera) Root Extract in Insomnia and Anxiety: A Double-blind, Randomized, Placebo-controlled Study

**DOI:** 10.7759/cureus.5797

**Published:** 2019-09-28

**Authors:** Deepak Langade, Subodh Kanchi, Jaising Salve, Khokan Debnath, Dhruv Ambegaokar

**Affiliations:** 1 Pharmacology, D.Y. Patil University School of Medicine, Navi Mumbai, IND; 2 Pharmacology, Vedantaa Institute of Medical Sciences, Palghar, IND; 3 Internal Medicine, Prakruti Hospital, Mumbai, IND; 4 Family Medicine, Prakruti Hospital, Mumbai, IND; 5 Pharmacology, D. Y. Patil University School of Medicine, Navi Mumbai, IND

**Keywords:** ashwagandha, insomnia, anxiety, sleep onset latency, actigraphy, wake after sleep onset, sleep efficiency

## Abstract

Introduction

Insomnia is a prevalent sleep disorder that can profoundly impact a person’s physical health and mental wellbeing. Most of the currently available drugs for insomnia exert adverse effects. Hence, alternative herbal therapies could be effective in treating insomnia. Ashwagandha, a proven “Rasayana” from ancient Ayurveda is having the required potential to treat insomnia.

Objective

To determine the efficacy and safety of Ashwagandha root extract in patients with insomnia and anxiety.

Methods

This was a randomized, double-blind, placebo-controlled study conducted at Prakruti Hospital, Kalwa, Maharashtra, India. A total of 60 patients were randomly divided into two groups: test (n = 40) and placebo (n = 20) in a randomization ratio of 2:1. Test product was a capsule containing highest concentration full-spectrum Ashwagandha root extract 300 mg, and the placebo was an identical capsule containing starch. Both treatments were given twice daily with milk or water for 10 weeks. Sleep actigraphy (Respironics Philips) was used for assessment of sleep onset latency (SOL), total sleep time (TST), sleep efficiency (SE) and wake after sleep onset (WASO). Other assessments were total time in bed (sleep log), mental alertness on rising, sleep quality, Pittsburgh Sleep Quality Index (PSQI), and Hamilton Anxiety Rating Scale (HAM-A) scales.

Results

Two patients, one from each group, did not complete study and the per-protocol dataset (n = 58) included 29 and 19 patients from test and placebo, respectively. The baseline parameters were similar in the two groups at baseline. The sleep onset latency was improved in both test and placebo at five and 10 weeks. However, the SOL was significantly shorter (p, 0.019) after 10 weeks with test [29.00 (7.14)] compared to placebo [33.94 (7.65)]. Also, significant improvement in SE scores was observed with Ashwagandha which was 75.63 (2.70) for test at the baseline and increased to 83.48 (2.83) after 10 weeks, whereas for placebo the SE scores changed from 75.14 (3.73) at baseline to 79.68 (3.59) after 10 weeks. Similarly, significant improvement in sleep quality was observed with test compared to placebo (p, 0.002). Significant improvement was observed in all other sleep parameters, i.e., SOL, SE, PSQI and anxiety (HAM-A scores) with Ashwagandha root extract treatment for 10 weeks.

Conclusion

Ashwagandha root extract is a natural compound with sleep-inducing potential, well tolerated and improves sleep quality and sleep onset latency in patients with insomnia at a dose of 300 mg extract twice daily. It could be of potential use to improve sleep parameters in patients with insomnia and anxiety, but need further large-scale studies.

## Introduction

Replenishing the regular health decays is the vital requirement of human health that is satisfied through sleep cycles. Sleep is a mandatory part of life that naturally rejuvenates us physiologically, biochemically, and at the cellular and molecular level as well. On average, more than 30% of human life is spent sleeping [1]. Apart from rejuvenation, sleep is directly associated with the proper function of the central nervous system, blood pressure maintenance, metabolism, catabolism, temperature regulation, memory consolidation and several other essential physiological functions [2]. In recent times, Insomnia or sleeplessness has become a common disorder that is affecting a large global population and impairing the general health and mental wellbeing. Insomnia is clinically characterized by difficulty in sleep onset or sleep maintenance or a combination of both and impairment of daily functionalities. The clinical criteria that designate insomnia are average sleep latency of more than 30 min, wakefulness after sleep onset of more than 30 min, sleep efficiency of less than 85% of total sleep time of fewer than 6.5 hours [3]. Insomnia often leads to fatigue, energy depletion, impairment in concentration and increased irritability. Investigations of etiological and pathophysiological factors since decades suggest that the onset and the progress of insomnia depend on multiple factors including genetic, cognitive and behavioral, physiological, biochemical, and environmental [4]. Impact of the cumulative effect of these factors often leads to other physiological and clinical complications such as autonomic nervous system (ANS) activation through altered heart rate, body temperature variation, varying metabolism, deviated function of the hormonal axis such as hypothalamic-pituitary-adrenal axis. The prognosis on onset and progress of insomnia is debatable and widely varies from case to case. Multiple factors can induce the disease condition including stress, anxiety, asthma, altering hormonal cycle, lifestyle modification and other disease conditions such as cancer [3]. Association of insomnia with chronic diseases, such as obesity, type 2 diabetes, cardiovascular disease, neurological issues including mood swings, increased mortality has been established as evidenced by available literature [5]. A survey suggests, more than 80% global population use plant extracts as medication for primary health care [6,7]. Ashwagandha found across India and south Asia, is a respected herb in traditional Indian Ayurveda, scientifically known as Withania somnifera (L.) Dunal. It is a member of the Solanaceae family [8]. The practitioners of Ayurveda regard Ashwagandha as a multipurpose and a valuable herb due to the extensive use of different plant parts to treat a variety of ailments. In Ayurveda, Ashwagandha has been utilized for centuries as a “Rasayana” and an adaptogen [9].

Many studies have been conducted on the root extracts of Ashwagandha. In vitro studies on Ashwagandha root extracts have demonstrated its neuroprotective, anti-inflammatory and chondroprotective effects [10,11]. Experiments using animal models indicated cardio-protective, immunomodulatory, anti-diabetic and neuroprotective effects of Ashwagandha root extracts [12-15]. Clinical studies conducted in various conditions suggest that Ashwagandha root extract improves sexual performances in both males and females, aids in reducing and managing stress and anxiety, improves memory and cognition in healthy adults and patients with bipolar disorder [16-20]. This multipurpose herb also increases muscle strength, muscle size and supports in muscle recovery [21].

In the present study, we have investigated the impact of Ashwagandha root extract powder compared to placebo on insomnia patients as a natural solution to the burgeoning problem of insomnia. The sleep-wake cycle was monitored using actigraphy devices which were simple and effective apart from analysis of sleep logs. Scientific literature evidence suggests that the sleep parameters estimated sleep actigraphy are sleep onset latency (SOL), total sleep time (TST), sleep efficiency (SE) and wake after sleep onset (WASO) [22]. Sleep actigraphy is a validated tool and has become a common practice along with standard sleep logs for sleep studies. A self-rated questionnaire, such as the Pittsburgh Sleep Quality Index (PSQI), was used to measure the quality and disturbances of sleep in adults. PSQI has seven components: subjective sleep quality, sleep latency, sleep duration, habitual sleep efficiency, sleep disturbances, use of sleeping medications and daytime dysfunction over the last month [23]. The Hamilton Anxiety Rating Scale (HAM-A) used in our study is another important first rating scale developed to measure the severity of anxiety symptoms. HAM-A consists of 14 elements and caters for both psychological and somatic symptoms [24].

## Materials and methods

Study objectives

**Figure 1:**
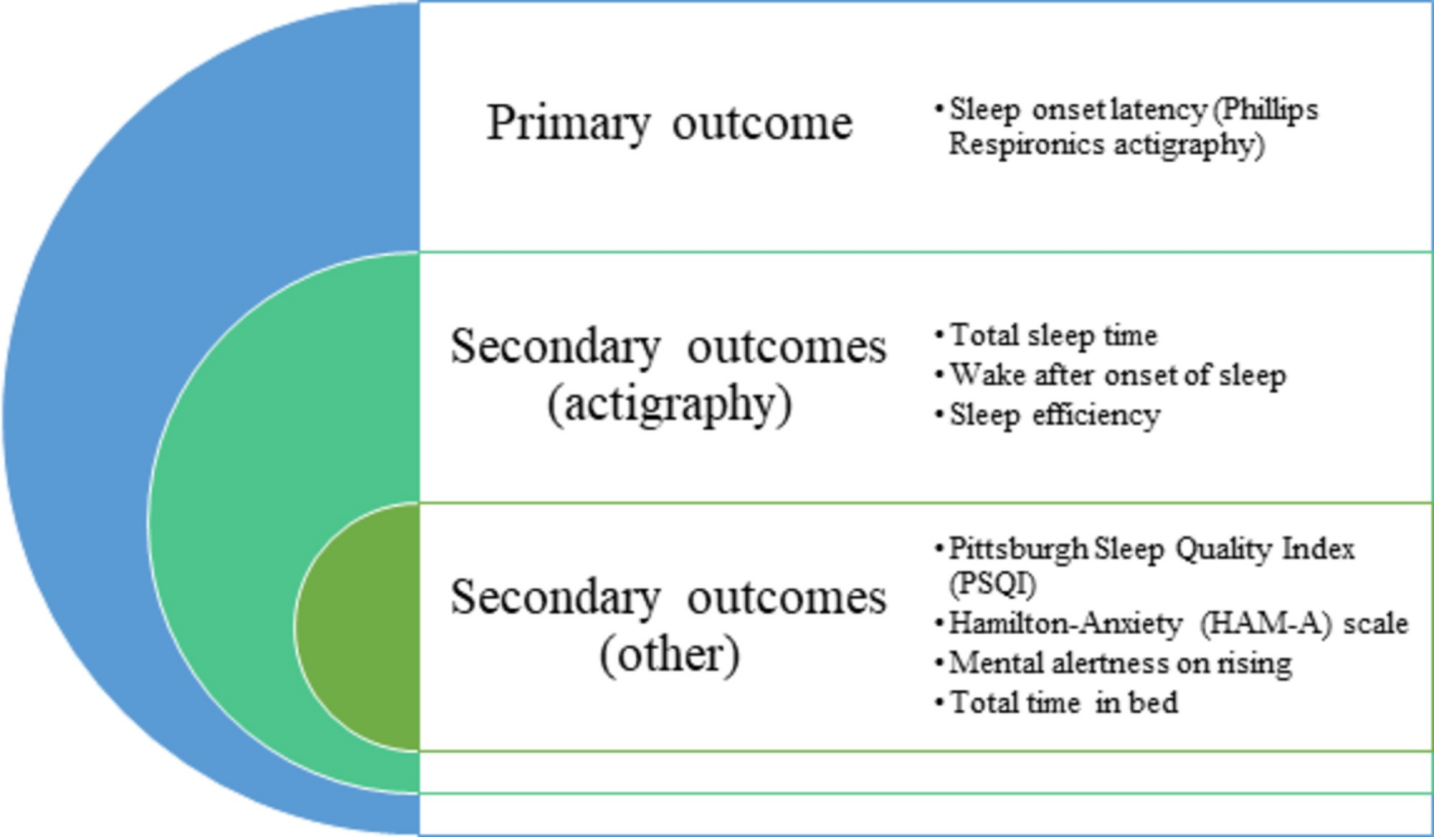
Study outcome measures

Primary objective was to compare the effects of Ashwagandha root extract and placebo on the SOL assessed by actigraphy. The secondary objectives include comparing the effects of Ashwagandha root extract versus placebo i) on the total sleep time (TST), Wake After Sleep Onset (WASO) and Sleep Efficiency (SE) assessed by actigraphy; ii) on sleep quality using the Pittsburgh Sleep Quality Index (PSQI), mental alertness on rising and sleep quality using Sleep logs; iii) on anxiety score using the Hamilton-Anxiety (HAM-A) scale (Figure 1).

Study design

This was a randomized, double-blind, placebo-controlled study conducted between November 2014 and March 2015. A total of 60 patients with insomnia were randomized in a 2:1 ratio to receive Ashwagandha root extract (KSM 66 capsule) or identical placebo for a period of 10 weeks. The study was conducted in accordance with the Helsinki Declaration (1989 amendment) and the study protocol was approved by an Institutional Ethics Committee (Reference number: ECR/66/Inst/MH/2013), Snehal Hospital, Thane dated 30 October 2014. Ethics Committee (EC) notifications were followed as per Good Clinical Practice (GCP) guidelines, issued by CDSCO and ethical guidelines for biomedical research on human patients, issued by Indian Council of Medical Research (ICMR). The Consolidated Standards Of Reporting Trials (CONSORT) guidelines for designing and reporting controlled trials were followed in this study. Written informed consent was obtained from all participants prior to the enrolment. Each patient was explained in detail about the study objective and the expected outcome before taking the consent.

Study participants

The study population included 60 participants aged between 18 and 60 years. Participants were selected from several outpatient clinics and invited to the study centre (Prakruti Hospital, Kalwa, Thane, Mumbai, India). Participants were informed about the study and were assessed by the principal investigator for eligibility based on the inclusion/exclusion criteria. Both male and female patients who were over 18 years of age and under 60 years of age and were diagnosed with insomnia based on Diagnostic and Statistical Manual (DSM‐IV) were included in the study. Patients were included when their body mass index was between 16.5 to 30 kg/m^2^. Another inclusion mandate was the ability of the patients to understand the sleep diary and their willingness to fill the sleep log dairy and follow other procedures required by the study protocol. Only those patients were considered who take more than 30 min to fall asleep and who reported subjective total sleep time of ≤ 6.5 hours per night. Moreover, habitual bedtime of between 8.30 pm and midnight was another inclusion criterion. Participants were excluded if they could not comply with the study protocol. The exclusion was also made if the subject had a history of seizures or significant head trauma, suffered from sleep disorder other than primary insomnia including restless leg syndrome and sleep apnea. Those who travelled across four or more time zones or worked on the night or rotating shifts in the previous seven days before study initiation were not considered. Subjects who had the plan to do work at night or follow a rotating shift during the study period and those who were taking any medications on a regular basis were excluded. Participants with clinically significant endocrine, metabolic, renal, hepatic, cardiovascular, gastrointestinal, respiratory, hematological or neurological illness and with any psychiatric disorders that can potentially result in insomnia were excluded. Individuals with substance abuse over a year, having alcohol and using tobacco were also considered ineligible.

Study procedures

In this 10-week long study, informed consent from each subject was obtained after screening and before enrolment. The participants were thoroughly screened for medical history, general physical conditions, and vital parameters. Once enrolled, the participants were assessed for the efficacy parameters for sleep assessments which include sleep actigraphy (Respironics Philips), PSQI, HAM-A scale, mental alertness on rising, sleep quality and sleep log [23,24]. The safety parameters were assessed based on any adverse events reported.

The test product used in this study was KSM-66 Ashwagandha capsule, a proprietary root-only extract (300 mg) of Ashwagandha (Withania somnifera Dunal) and is manufactured by Ixoreal Biomed Inc., Los Angeles, California, USA. The product is light yellow in colour with the highest concentration of withanolides (5%>). The placebo was a starch powder which was identical to test capsules. Both the test product and the placebo were coated in a capsule of exact shape, size, and colour. The batch number of the test product was KSM/14/270 which was manufactured in August 2014.

Capsules containing either Ashwagandha root extract (test) or placebo were used for intervention. Participants were instructed to take one capsule twice daily with milk or water for a period of 10 weeks. All the subjects were evaluated at the screening, baseline, 5th week and 10th week with the outcome measures. Physical examination and vital parameters of study participants were monitored during each visit. General examination and detailed monitoring of respiratory systems, cardiovascular systems, musculoskeletal system, digestive system and nervous system of each subject were done. In addition, measurements of systolic blood pressure, diastolic blood pressure, pulse rate, respiratory rate and body temperature of the subjects were noted in every visit.

Outcome measures of the study were the actigraphy parameters (sleep onset latency, total sleep time, wake after the onset of sleep), PSQI and HAM-A scores, mental alertness and sleep quality and total time in bed (TIB) (Figure 1).

Sleep actigraphy

Monitoring of rest and activity was performed using sleep actigraphy, a non-invasive, sensor-based procedure where the wearable part of the device can be placed on the wrist for the entire study duration to record movement. The procedure is validated against gold standard Nocturnal Polysomnogram.

SOL, TST, WASO and SE were the parameters assessed using the actigraphy. Clinically, the conversion period from full wakefulness to light sleep is termed as SOL. This stage often includes the initial stage of non-rapid eye movement sleep (NREMS). TST is referred to as the actual sleep time in a sleep period. Total sleep time is the cumulative outcome of the rapid eye movement sleep (REMS) and NREMS in an entire sleep period. WASO is the duration, a person remains awake after the defined onset of the sleep. Essentially, the estimation of WASO considers the specific wakefulness phase that is devoid of the duration prior to the sleep onset. SE refers to the actual time spent for sleep (total sleep) in comparison to the overall time spent in bed.

The computed data stored in the actigraphy devices were transferred to a computer for display and analysis of the period of wakefulness and sleep for all the subjects. Minimum three days continuous use of the actigraph was recommended for the enrolled patients to obtain a comprehensive view of the patient’s sleep-wake pattern.

Total time in bed

Total time in bed was assessed by the individual sleep log, which was a daily, written record of the individual’s sleep-wake pattern. The log contained information on the time of retiring and arising, time in bed, estimated total sleep period, number and duration of sleep interruptions, quality of sleep, daytime naps, use of medications or caffeine beverages per day and nature of waking activities.

Pittsburgh sleep quality index (PSQI)

The PSQI is a self-administered questionnaire resulting to a subjective measure of sleep quality and patterns through estimating the outcome of seven components of sleep quality, i.e., subjective sleep quality, sleep latency, sleep duration, habitual sleep efficiency, sleep disturbances, use of sleep-promoting medications, and daytime dysfunction over the period of a month [23]. The summed up global score of the seven components estimates the patient’s sleep quality that varies from 0 to 21. Higher the scores, worse the sleep quality.

Hamilton anxiety rating scale (HAM-A)

HAM-A is represented by 14 items [24]. Every item individually encompasses a group of symptoms. This scale estimates both psychic anxiety and somatic anxiety including mental agitation and psychological distress; and physical complaints related to anxiety, respectively. Every item estimation score ranges between 0 and 4; higher the score, severe the situation. The total score ranges from 0 to 56. In this scale, score <17 indicates mild severity, 18-24 represents mild to moderate severity, and 25-30 refers moderates to severe condition.

Mental alertness on rising

After waking up in the morning, the mental alertness was estimated through a three-point scale to assess the alertness as perceived by the patient where 1 refers to alert, 2 refers to slightly drowsy and 3 signifies extremely drowsy.

Sleep quality

Overall sleep quality was assessed using a seven-point scale as perceived by the patient after waking up in the morning. The scoring was considered as follows: 1 = Excellent, 2 = Very Good, 3 = Good, 4 = Fair, 5 = Poor, 6 = Very Poor and 7 = Extremely Poor.

Safety assessment

Clinical safety and tolerability were assessed based on the adverse events reported by the patients during the follow-up or during the clinical evaluation. Adverse events were recorded, along with their severity, duration and relationship to study drug.

Sample size and randomization

This was an exploratory study and it was planned to enroll 60 patients in the study. The sample size was not based on any statistical calculation or assumption. The enrolled 60 patients were randomized in a 2:1 ratio. Thus, 40 patients were considered in the experimental group (test product Ashwagandha capsules) and 20 patients were randomized to the control group (placebo group).

The randomization was done using PC-based software (Rando 1.2 R.Raveendran, 2004). The test and control products were packed in a way that the experimental and control study materials were identical in appearance. The packs were coded to conceal the nature of the drugs and the label contained the patient serial number which was the study ID. Once the participants were enrolled, they were provided with the study medication pack having the corresponding serial number. The randomization codes were provided in a separate sealed envelope for each participant and the envelope was opened by the investigator after the subjects were enrolled and received the serial number. Participants were not aware of their group assignments in this trial. Researchers and clinicians were blinded as well.

Statistical analysis

All the enrolled patients’ data were collected as determined in the study protocol, i.e., during baseline estimation, at the 5th week and at the 10th week. Data were analyzed according to their randomized group, regardless of compliance with the treatment or any other deviation from protocol. All statistical analyses were done using windows based program Stata IC/13 (StataCorp LLC, USA). The analysis was conducted in both intent-to-treat (ITT) and per-protocol (PP) datasets. The obtained analysis outcome and scores are represented here as means with 1 standard deviation (SD) and 95% confidence intervals (CI). Baseline scores were compared to the post-treatment scores for the different scales using the Friedman test (repeat measures) within the group. The two groups were compared for differences using the one way analysis of variance (ANOVA). All testing was done using two-tailed tests at alpha 0.05.

Paired comparisons were done within each group for comparison of baseline scores with follow-up scores (five weeks and 10 weeks). Non-parametric tests for mental alertness and sleep quality were compared between data collections intervals using Kruskal-Wallis test and chi-square test.

## Results

**Figure 2:**
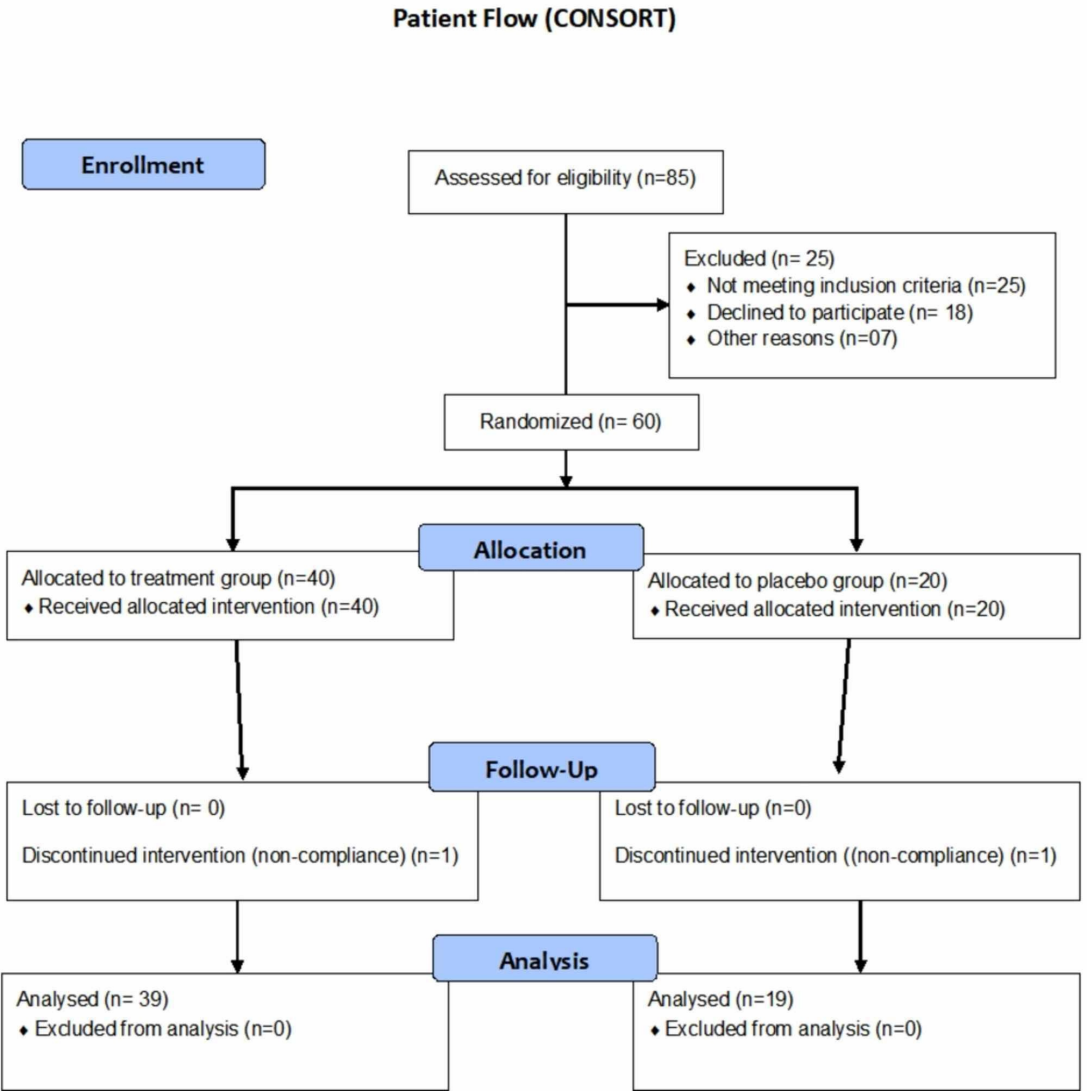
CONSORT flow representation of the patient enrollment, allocation, follow-up and analysis for this study CONSORT: Consolidated Standards Of Reporting Trials

A total of 85 participants were screened and assessed for eligibility initially, out of which, 25 participants failed eligibility criteria (Figure 2). The remaining 60 participants underwent randomization and were allocated to two groups: experimental and control group in a ratio of 2:1. Two participants (one from each group) withdrew from the study due to non-compliance to actigraphy (Figure 2). The analysis was continued using the data for the remaining 58 participants through per-protocol (PP) analysis. Table 7 (appendices) represents the study schedule followed.

Demography

Demography of the patients is presented in Table 1.

**Table 1:** Demography of patients enrolled BMI: Body mass Index

	Ashwagandha			Placebo			ANOVA	
	Mean	SD	95% C.I.	Mean	SD	95% C.I.	F	p
Intent-to-treat (ITT) dataset (n = 60)								
N	40			20				
Age (yrs.)	38.83	5.00	37.23 - 40.42	40.00	6.21	37.09 - 42.90	0.626	0.432
BMI (kg/sq.m)	26.91	3.42	25.81 - 28.00	25.89	6.02	23.07 - 28.71	0.695	0.408
Per-protocol (PP) dataset (n = 48)								
N	39			19				
Age (yrs.)	38.97	4.97	37.36 - 40.59	40.05	6.37	36.98 - 43.12	0.498	0.483
BMI (kg/sq.m)	26.87	3.46	25.75 - 28.00	25.28	5.50	22.62 - 27.93	1.834	0.181
		No.	%		No.	%		p
Gender (M/F)								
ITT dataset		31/9	77.5%/22.5%		16/4	80.0%/20.0%		0.8261
PP dataset		31/8	79.5%/20.5%		4	78.9%/21.1%		0.9263

The two groups were similar with respect to the age, body mass index (BMI) and gender distribution at baseline.

Sleep parameters and actigraphy outcomes

Sleep parameters were recorded using an actigraphy watch on screening, baseline, week 5 and week 10 (Table 2) and all the timings are expressed in minutes. At baseline, the mean (SD) SOL scores and mean WASO scores for the test group were 41.61 (6.84) and 42.69 (14.66), respectively. For the control group, the values were 41.94 (6.98) and 44.00 (12.43), correspondingly. After 10 weeks, the assessed scores for SOL and WASO were 29.00 (7.14) and 33.05 (14.36), respectively, recorded for the experimental group. For the control group, it was 33.94 (7.65) and 40.00 (12.26).

**Table 2:** Sleep parameters with actigraphy in patients who completed study

	Ashwagandha (n = 39)		Placebo (n = 19)		ANOVA	
	Mean (SD)	95% C.I.	Mean (SD)	95% C.I.	F	Sig.
Sleep onset latency (min)						
Baseline	41.62 (6.84)	39.40 - 43.83	41.95 (6.99)	38.58 - 45.32	0.030	0.864
5 weeks	35.18 (7.04)	32.90 - 37.46	38.11 (7.01)	34.73 - 41.48	2.215	0.142
10 weeks	29.00 (7.15)	26.68 - 31.32	33.95 (7.66)	30.26 - 37.64	5.847	0.019
Total sleep time (min)						
Baseline	261.77 (34.78)	250.49 - 273.04	263.00 (42.72)	242.41 - 283.59	0.014	0.907
5 weeks	290.92 (35.61)	279.38 - 302.47	276.16 (43.46)	255.21 - 297.11	1.898	0.174
10 weeks	311.62 (35.81)	300.01 - 323.22	292.37 (42.47)	271.90 - 312.84	3.265	0.076
Wake after sleep onset (min)						
Baseline	42.69 (14.67)	37.94 - 47.45	44.00 (12.43)	38.01 - 49.99	0.112	0.740
5 weeks	38.21 (14.42)	33.53 - 42.88	41.84 (12.19)	35.96 - 47.72	0.895	0.348
10 weeks	33.05 (14.36)	28.40 - 37.71	40.00 (12.27)	34.09 - 45.91	3.276	0.076
Total time in bed (min)						
Baseline	346.08 (44.48)	331.66 - 360.50	348.95 (47.81)	325.90 - 371.99	0.051	0.823
5 weeks	364.31 (45.23)	349.65 - 378.97	356.11 (48.21)	332.87 - 379.34	0.403	0.528
10 weeks	373.67 (45.04)	359.07 - 388.27	366.32 (46.85)	343.74 - 388.90	0.332	0.567
Sleep efficiency						
Baseline	75.63 (2.70)	74.76 - 76.51	75.14 (3.73)	73.34 - 76.94	0.322	0.573
5 weeks	79.91 (2.67)	79.04 - 80.77	77.35 (3.56)	75.64 - 79.07	9.356	0.003
10 weeks	83.49 (2.84)	82.57 - 84.41	79.68 (3.59)	77.95 - 81.41	19.265	<0.0001

After completion of the study at 10th week, there was a significant decrease in SOL, WASO scores in the experimental group relative to the control group. The impact of the treatment is evident from the result.

In the case of total sleep time, the mean value at the baseline was 261.76 (34.78) for the experimental group and 263.00 (42.72) for the control group. At the end of the study, the scores recorded were 311.61 (35.80) for the experimental group and 292.36 (42.46) for the control group, respectively. In contrast to the SOL and WASO results, a significant increase was recorded in TST, TIB, and SE in the experimental group in comparison to the control group. The ANOVA results of the sleep parameters between the groups and within the groups are presented in Table 8 (appendices).

The outcome suggests that the mean total sleep time significantly increased for the experimental group after treatment. In continuation of the above observations, the mean change in the total time in bed (TIB) was noted higher in the experimental treatment group compared to the placebo (control) counterparts. At baseline, the total time in bed was 346.08 (44.48) for the experimental group and 348.94 (47.81) for control. At the end of the study (10th week), the obtained total time in bed was recorded as 373.66 (45.03) for the experimental group, and 366.31 (46.84) for the placebo or control group. Mean SE at baseline noted was 75.63 (2.70) for the experimental group and 75.14 (3.73) for the control group. The documented values at the end of the study were 83.48 (2.83) for the experimental group and 79.68 (3.58) for the control group, respectively. The difference in mean change between the two treatment groups was statistically significant (P < 0.001).

A comparative analysis was conducted between the placebo and the test group to understand the participants’ performance differences among various sleep parameters such as SOL, TST, TIB, and sleep efficiency as represented in Table 2. The standard deviation values between the baseline measurements of the parameters and week 5 and week 10 suggest the significant differences along with the significant impact of the test product at the end of the study. Excellent improvement was observed for TST, TIB and overall sleep efficiency for the experimental group.

**Figure 3:**
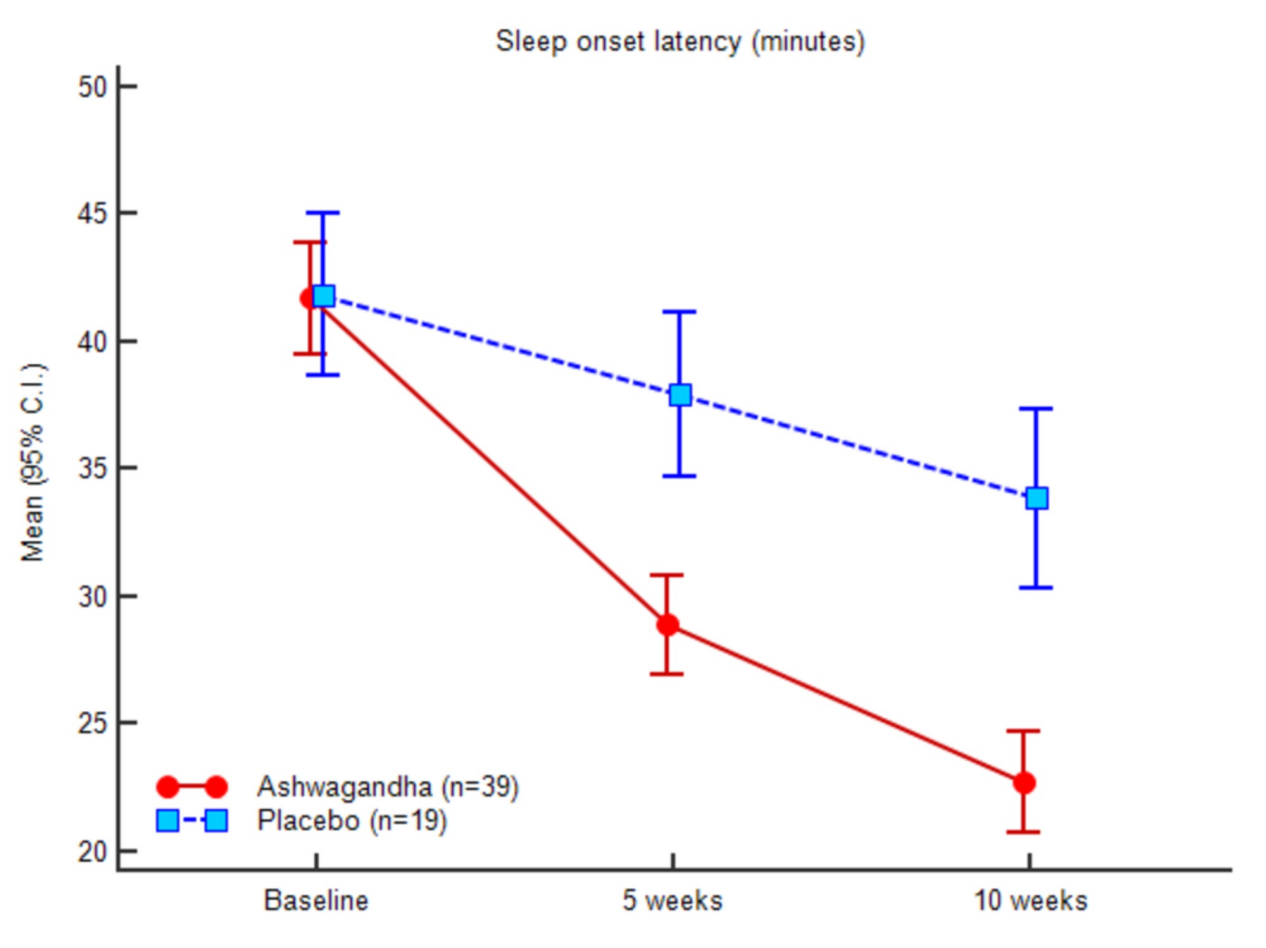
Sleep onset latency (minutes)

**Figure 4:**
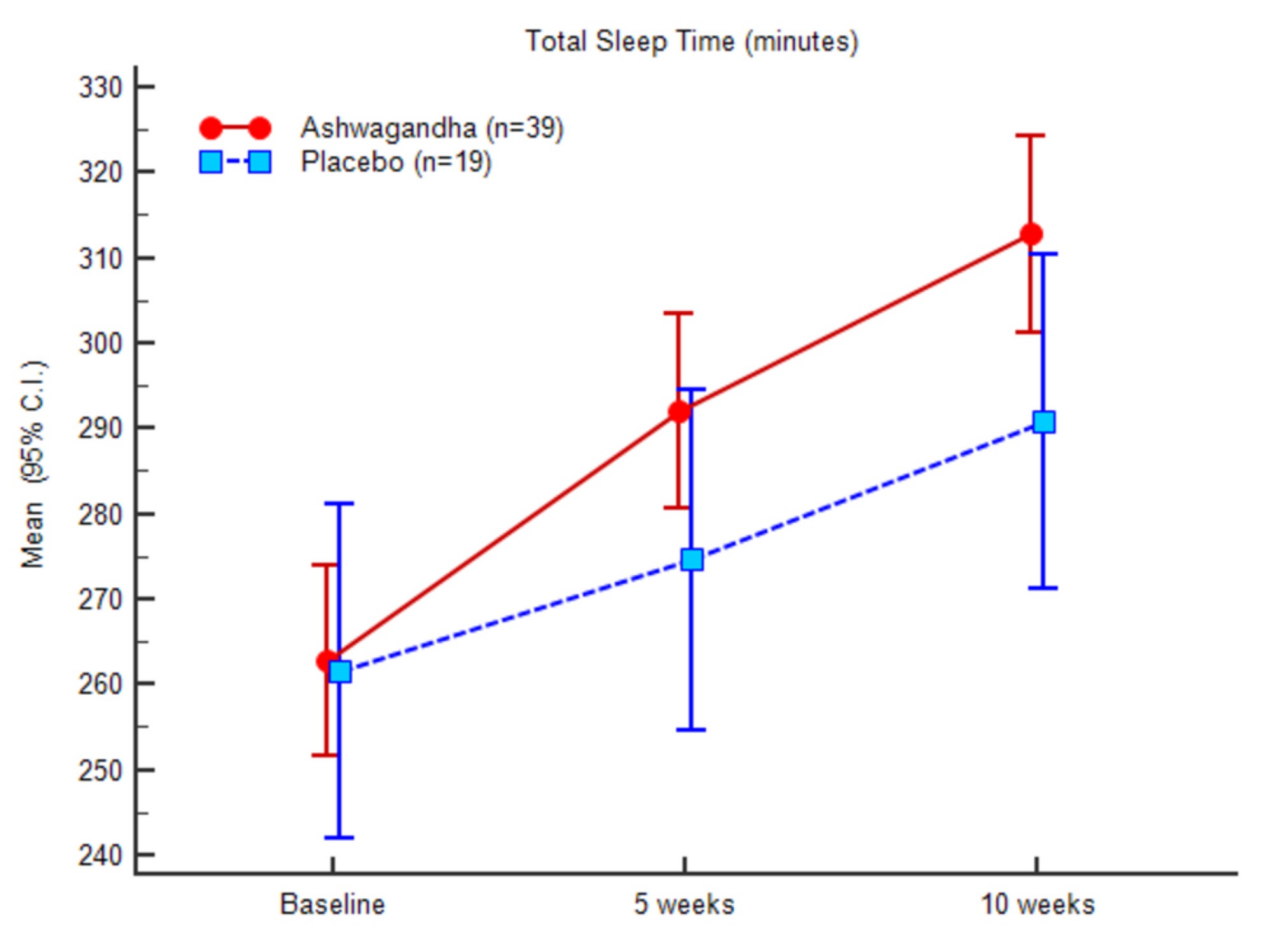
Total sleep time (minutes)

**Figure 5:**
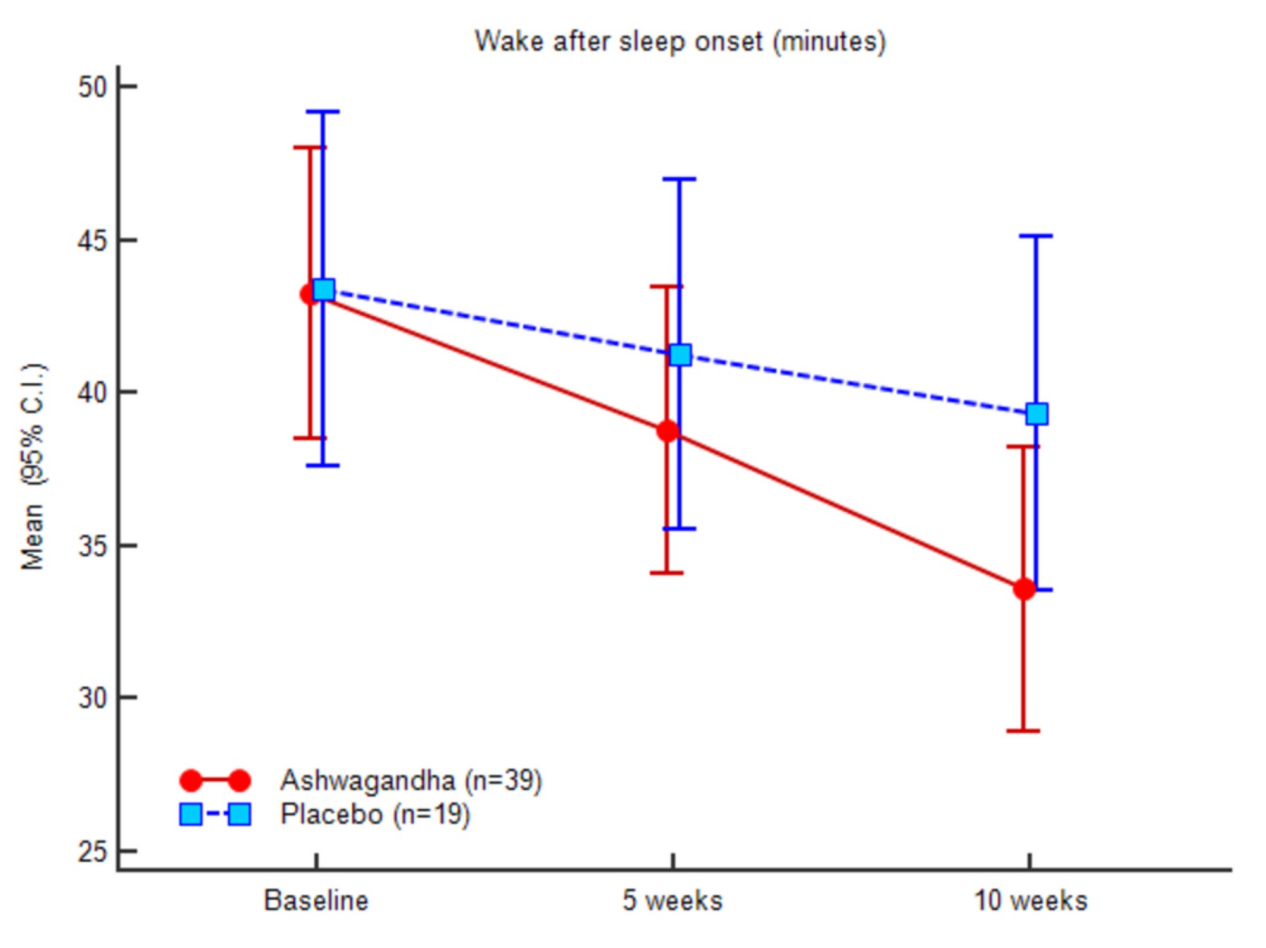
Wake after sleep onset (minutes)

**Figure 6:**
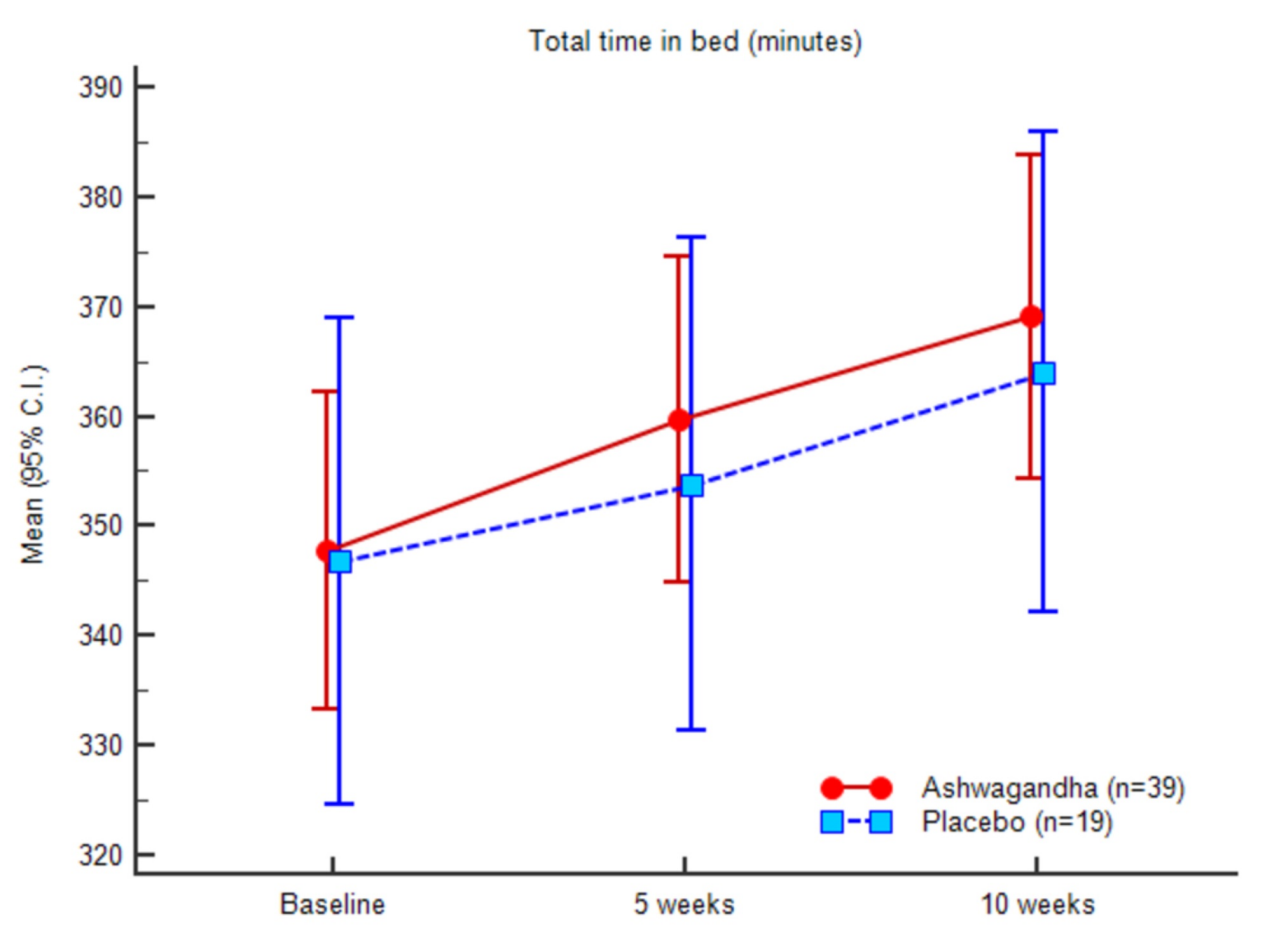
Total time in bed (minutes)

**Figure 7:**
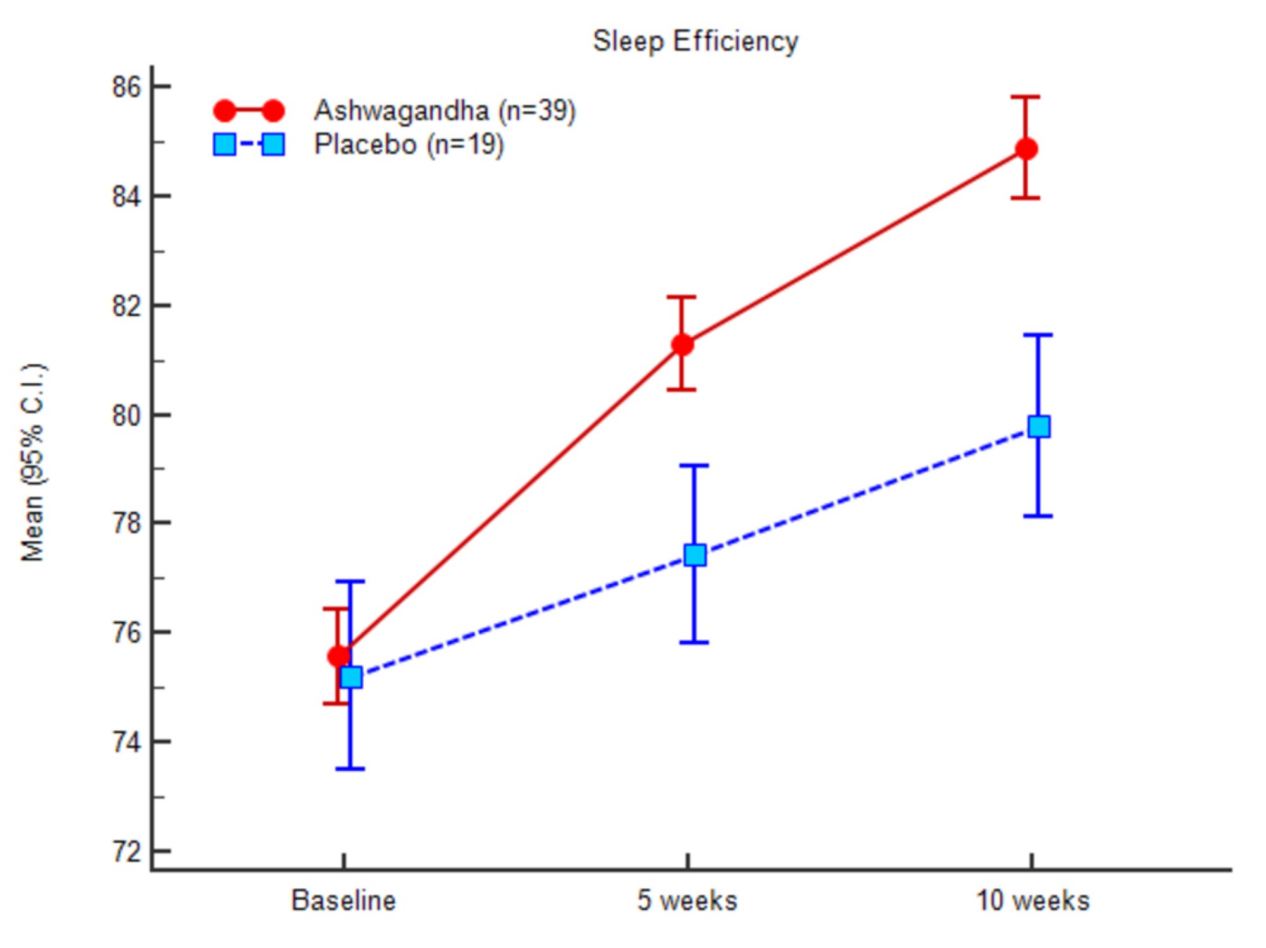
Sleep efficiency (actigraphy)

Figures 3-7 represent the trend observed throughout the study for the parameters considered, i.e., SOL, TST, WASO, TIB and SE, respectively.

PSQI outcomes

**Figure 8:**
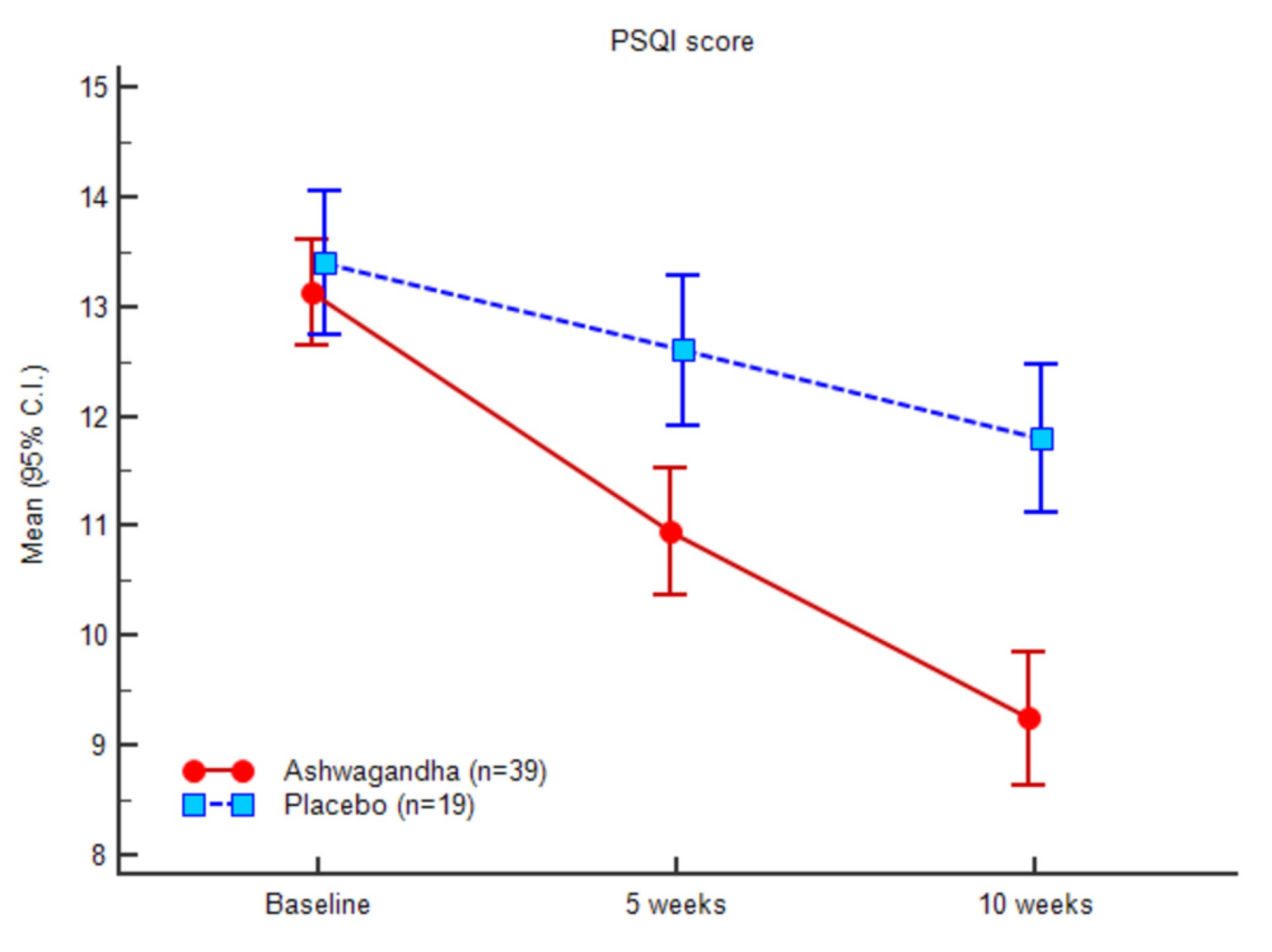
Pittsburgh sleep quality index (PSQI) scores

Greater PSQI score changes for the test group was documented at the end of the study compared to the baseline values. The mean PSQI score at the baseline was 13.07 (1.51) and 9.15 (1.82) at the end of the study for the test group. In comparison, the mean PSQI for the control group recorded at baseline was 13.47 (1.38), and at the end of the study, it was 11.8 (1.46). Table 3 and Figure 4 demonstrate the PSQI scores of both the groups at baseline, week 5 and week 10. There was a significant decrease in the PSQI scores of the experimental group when compared to the control group. The gradual decrease in the PSQI scores is prominent in Figure 8 for both the groups. The comparative measure further projects that the treatment with the Ashwagandha root extract powder depicted better outcome in comparison to the placebo group.

**Table 3:** Sleep quality (PSQI) scores

	Ashwagandha (n = 39)		Placebo (n = 19)		ANOVA	
	Mean (SD)	95% C.I.	Mean (SD)	95% C.I.	F	Sig.
Baseline	13.08 (1.51)	12.59 - 13.57	13.47 (1.39)	12.80 - 14.14	0.927	0.340
5 weeks	10.85 (1.71)	10.29 - 11.40	12.63 (1.50)	11.91 - 13.35	15.054	<0.0001
10 weeks	9.15 (1.83)	8.56 - 9.75	11.84 (1.46)	11.14 - 12.55	31.221	<0.0001

Anxiety outcomes

**Figure 9:**
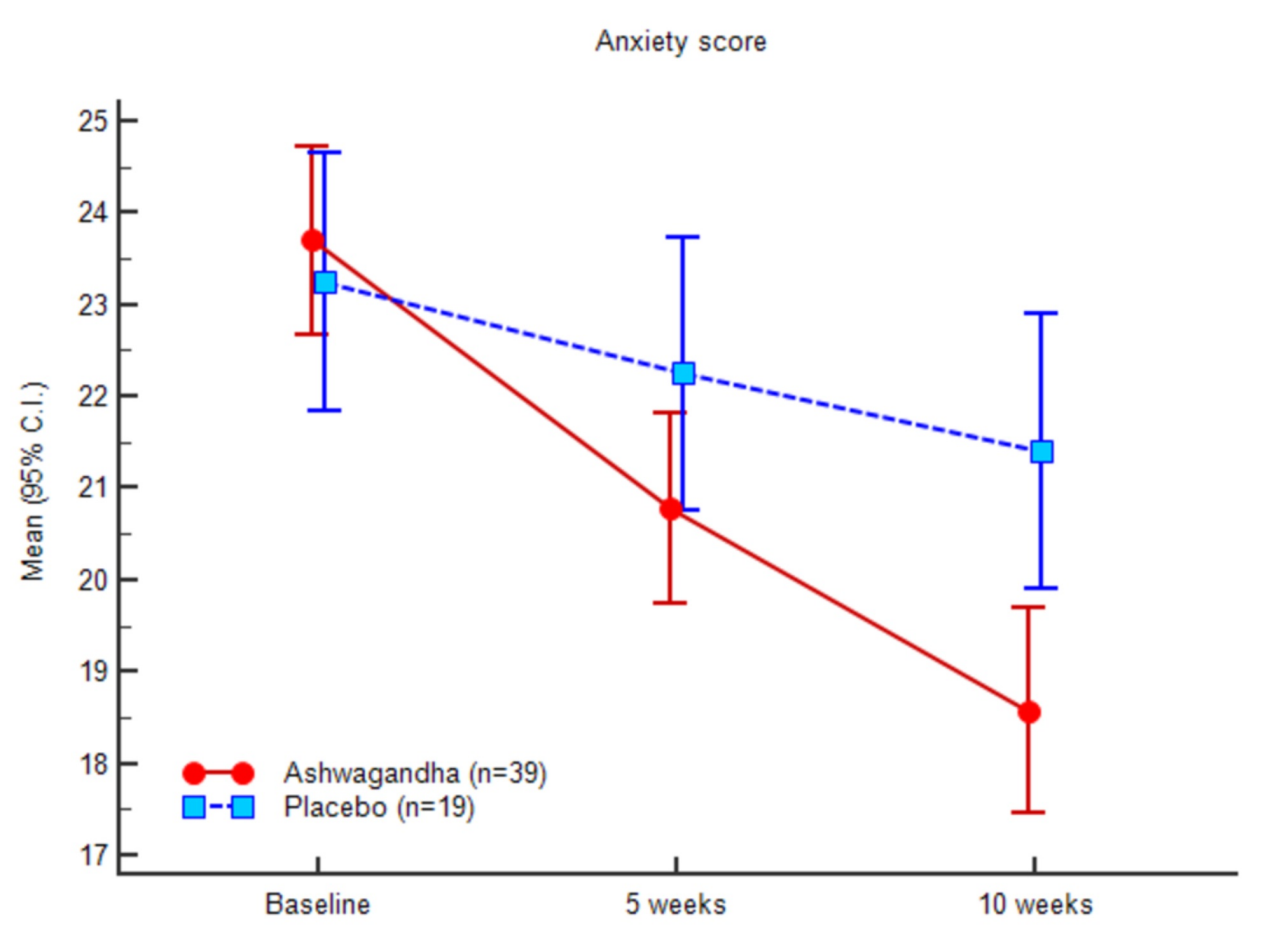
Anxiety (HAM-A) scores

The Hamilton Anxiety Rating Scale was used to determine anxiety. There was a significant decrease in the HAM-A score for the experimental group when compared with the control group. At baseline, the HAM-A scores were 23.58 (3.15) and 23.42 (3.00) for the experimental and control groups, respectively. At week 10, the HAM-A scores were 18.48 (3.47) and 21.52 (3.22), respectively. Table 4 and Figure 9 demonstrate the HAM-A scores of both the groups at baseline, at week 5 and at week 10. Treatment with Ashwagandha resulted in comparative lower values than that of the placebo counterparts.

**Table 4:** Anxiety (HAM-A) scores

	Ashwagandha (n = 39)		Placebo (n = 19)		ANOVA	
	Mean (SD)	95% C.I.	Mean (SD)	95% C.I.	F	Sig.
Baseline	23.59 (3.15)	22.57 - 24.61	23.42 (3.01)	21.97 - 24.87	0.038	0.847
5 weeks	20.69 (3.24)	19.64 - 21.74	22.37 (3.22)	20.82 - 23.92	3.437	0.069
10 weeks	18.49 (3.48)	17.36 - 19.61	21.53 (3.22)	19.97 - 23.08	10.222	0.002

Mental alertness on rising and sleep quality

Excellent sensory awareness with acceptable active attention is considered as mental awareness. Lower alertness is noted due to exhaustion, lack of energy, drowsiness, etc. whereas the alternative conditions may allow a person to be most active. The mental alertness on rising for the patients considered in this study was scaled as alert (score 1), slightly drowsy (score 2) and extremely drowsy (score 3). The data for mental alertness is presented in Table 5.

**Table 5:** Mental alertness on rising (% of pts.)

	Ashwagandha (n = 39)		Placebo (n = 19)		Mann-Whitney ‘U’ test	
	No.	%	No.	%	^2^	p
Baseline						
Alert	4	10.3%	4	21.1%	0.019	0.890
Slightly Drowsy	23	59.0%	8	42.1%		
Extremely Drowsy	12	30.8%	7	36.8%		
5 weeks						
Alert	25	64.1%	10	52.6%	0.910	0.340
Slightly Drowsy	11	28.2%	6	31.6%		
Extremely Drowsy	3	7.7%	3	15.8%		
10 weeks						
Alert	27	69.2%	10	52.6%	1.839	0.175
Slightly Drowsy	11	28.2%	7	36.8%		
Extremely Drowsy	1	2.6%	2	10.5%		

More number of patients with Ashwagandha were alert as compared to placebo at five weeks (p, 0.340) and 10 weeks (p, 0.175). A gradual improvement from the baseline to the final assessment in the tenth week was noted in both groups.

Sleep quality was assessed through the scale as mentioned in the method section. The statistical outcome of the non-parametric hypothesis testing on the per-protocol patients is presented in Table 6. There is a gradual improvement in the sleep quality with Ashwagandha treated group whereas the placebo group does not show improvement. Better improvement with the test treatment versus placebo can be seen as the p-value has reduced over the study period (baseline, p = 0.697; five weeks, p = 0.281 and at 10 weeks, p = 0.002).

**Table 6:** Sleep quality (% of pts.)

	Ashwagandha (n = 39)		Placebo (n = 19)		Mann-Whitney ‘U’ test	
	No.	%	No.	%	^2^	p
Baseline						
Fair	1	2.6%	1	5.3%	0.152	0.697
Poor	16	41.0%	6	31.6%		
Very Poor	15	38.5%	8	42.1%		
Extremely Poor	7	17.9%	4	21.1%		
5 weeks						
Very Good	1	2.6%	0	0.0%	1.162	0.281
Good	8	20.5%	1	5.3%		
Fair	10	25.6%	6	31.6%		
Poor	13	33.3%	8	42.1%		
Very Poor	6	15.4%	4	21.1%		
Extremely Poor	1	2.6%	0	0.0%		
10 weeks						
Excellent	1	2.6%	0	0.0%	9.481	0.002
Very Good	8	20.5%	0	0.0%		
Good	10	25.6%	2	10.5%		
Fair	11	28.2%	6	31.6%		
Poor	6	15.4%	8	42.1%		
Very Poor	3	7.7%	3	15.8%		

Safety outcomes

None of the patients reported any adverse events during the study period.

## Discussion

Insomnia has become a global concern due to the modern urban lifestyle and other socio-economic changes. The recent global survey suggests that insomnia has become a common problem with complaints of associated stress, sleep apnea, hormonal imbalance as prime reasons. Often, chronic fatigue, endocrinological issues, energy depletion, lack of attention are the initial outcomes of chronic insomnia followed by illness associated with mild or severe disease conditions including blood pressure, depression, renal disease, cognitive impairment, diabetes, cardiovascular diseases, and others. Reduction of average sleep hours and increase in daily stress serves as the initial trigger for insomnia. Often, insomnia becomes clinically challenging to identify and characterize, an inspection of the clinical history can aid in understanding the factors contributing to the disease condition, behavioral treatment was also recommended by Buysse [1].

The present treatment line pertaining to insomnia is discrete and multiple options have been attempted by the medical practitioners to counter the situation. The range of treatment varies from conventional medication such as neuropsychiatric drugs, cognitive behavioral therapy for insomnia (CBT-I), precision medicine, and alternative medicinal approaches. Earlier, a number of measures to negotiate chronic insomnia have been attempted including nonpharmacological treatment, bedtime restriction, use of receptor antagonists in relation to orexin system for sleep-onset insomnia [25,26]. None of these strategies were found to provide an ultimate solution for the chronic or sleep onset insomnia conditions. Conventional sleep medications are associated with rebound insomnia and withdrawal whereas elderly patients often experience decline in cognition. Thus, alternative medicine may provide a reasonable solution in this regard. Different methods of using alternative medicine including personalized therapeutic trials applying the herbal products have been recommended [27].

Among various alternative approaches attempted to tackle insomnia, herbal therapies have shown promises. A number of herbal sources have shown reasonable outcome including Ashwagandha. Application of Ashwagandha along with traditional Ayurvedic system also provided the desired result [28]. Proven efficacy and safety of the use of Ashwagandha through clinical studies have been documented earlier including reproductive issues, stress and anxiety, cardiorespiratory endurance and in other ailments [16,18,29].

The present study is the first report in the direction of clinical research where the scientific clinical study is conducted to understand the effect of Ashwagandha root extract on sleep quality in the considered patient population. Various sleep parameters were included in this study along with the level of anxiety in a 10-week treatment period, and the outcome was compared with a placebo via a randomized, double-blind, controlled clinical trial. Significant improvement of different components of sleep quality, sleep onset latency, and reduced anxiety was observed while using Ashwagandha root extract in participants for the insomnia patients compared to the placebo group. Kaushik et al. reported that the active ingredient of Ashwagandha leaves such as tryethylene glycol can induce sleep in mice through reducing the NREM sleep onset latency period [30].

The present 10 weeks study reports the demonstrated impact of the significant improvement in the sleep parameters SOL, SE, PSQI and anxiety parameters HAM-A. The obtained differences with the placebo group were found statistically significant, suggesting a substantial effect of Ashwagandha in improving the sleep and reducing the anxiety of the subjects.

Limitations

The accommodated sample size of the study was large enough to extract statistical inference but was not enough to draw a general conclusion on the impact of the supplement used. A large randomized controlled study may yield further information related to the population-wide safety and efficacy of the herbal product. Furthermore, though the enrolled subjects had sleep issues, none had any psychological illness or any other systemic illness. Studies using a wider set of clinical backgrounds would be illuminating. Similarly, the study duration should be increased to understand the long-term effect of Ashwagandha in patients with insomnia and anxiety.

## Conclusions

Available conventional therapies of insomnia are known to develop drug dependency and exert side effects. Ashwagandha extract is a natural compound with sleep-inducing potential is well tolerated and improves sleep quality and sleep onset latency in patients with insomnia at a dose of 300 mg extract twice daily. It could be a potential candidate for treatment of insomnia and anxiety.

## Appendices

**Table 7:** Study schedule

Visit	V1	V2	V3	V4
Day	Screening Day 3	Baseline Day 0	5 Weeks ± 2 Days	10 Weeks ± 2 Days
Informed consent	√	X	X	X
Demographics and Medical history	√	X	X	X
Screening and Enrolment	√	X	X	X
Physical examination & vital parameters	√	√	√	√
Study Medication Dispensing	X	√	X	X
Actiwatch (sleep actigraphy)	√	√	√	√
Pittsburgh Sleep Quality Index (PSQI)	√	√	√	√
Hamilton Anxiety Rating Scale (HAM-A)	√	X	√	√
Mental alertness on rising	√	X	√	√
Sleep quality	√	X	√	√
Sleep Log	X	√	√	√
Medication Compliance	X	X	√	√
Adverse Events	√	√	√	√
Concomitant Illness	√	X	X	X
Concomitant medication	√	√	√	√
Study Completion/Termination	X	X	X	√

**Table 8:** ANOVA table for sleep parameters in PP dataset (n = 58) WASO: Wake after sleep onset; ANOVA: Analysis of variance; PP: Per-protocol.

		Sum of Squares	df	Mean Square	F	Sig.
Sleep Onset Latency (min.) - Baseline	Between Groups	1.408	1	1.408	.030	0.864
	Within Groups	2656.178	56	47.432		
	Total	2657.586	57			
Sleep Onset Latency (min.) - 5 weeks	Between Groups	109.363	1	109.363	2.215	0.142
	Within Groups	2765.533	56	49.385		
	Total	2874.897	57			
Sleep Onset Latency (min.) - 10 weeks	Between Groups	312.708	1	312.708	5.847	0.019
	Within Groups	2994.947	56	53.481		
	Total	3307.655	57			
Total Sleep Time(min.) - Baseline	Between Groups	19.353	1	19.353	.014	0.907
	Within Groups	78826.923	56	1407.624		
	Total	78846.276	57			
Total Sleep Time (min.) - 5 weeks	Between Groups	2785.273	1	2785.273	1.898	0.174
	Within Groups	82183.296	56	1467.559		
	Total	84968.569	57			
Total Sleep Time (min.) - 10 weeks	Between Groups	4732.762	1	4732.762	3.265	0.076
	Within Groups	81179.652	56	1449.637		
	Total	85912.414	57			
WASO (min.) - Baseline	Between Groups	21.847	1	21.847	.112	0.740
	Within Groups	10958.308	56	195.684		
	Total	10980.155	57			
WASO (min.) - 5 weeks	Between Groups	168.994	1	168.994	.895	0.348
	Within Groups	10576.885	56	188.873		
	Total	10745.879	57			
WASO (min.) - 10 weeks	Between Groups	616.878	1	616.878	3.276	0.076
	Within Groups	10545.897	56	188.320		
	Total	11162.776	57			
Time in Bed (min.) - Baseline	Between Groups	105.266	1	105.266	.051	0.823
	Within Groups	116343.717	56	2077.566		
	Total	116448.983	57			
Time in Bed (min.) - 5 weeks	Between Groups	859.558	1	859.558	.403	0.528
	Within Groups	119558.097	56	2134.966		
	Total	120417.655	57			
Time in Bed (min.) - 10 weeks	Between Groups	690.349	1	690.349	.332	0.567
	Within Groups	116588.772	56	2081.942		
	Total	117279.121	57			
Sleep Efficiency (SE) - Baseline	Between Groups	3.034	1	3.034	.322	0.573
	Within Groups	527.412	56	9.418		
	Total	530.446	57			
Sleep Efficiency (SE) - 5 weeks	Between Groups	83.290	1	83.290	9.356	0.003
	Within Groups	498.508	56	8.902		
	Total	581.798	57			
Sleep Efficiency (SE) - 10 weeks	Between Groups	184.805	1	184.805	19.265	<0.0001
	Within Groups	537.192	56	9.593		
	Total	721.997	57			
